# Nonlocal electron correlations in an itinerant ferromagnet

**DOI:** 10.1038/s41467-018-05960-5

**Published:** 2018-09-13

**Authors:** Christian Tusche, Martin Ellguth, Vitaliy Feyer, Alexander Krasyuk, Carsten Wiemann, Jürgen Henk, Claus M. Schneider, Jürgen Kirschner

**Affiliations:** 1 Forschungszentrum Jülich, Peter Grünberg Institut (PGI-6), 52425 Jülich, Germany; 2 Fakultät für Physik, Universität Duisburg-Essen, 47057 Duisburg, Germany; 3 Max-Planck-Institut für Mikrostrukturphysik, Weinberg 2, 06120 Halle, Germany; 4 Institut für Physik, Martin-Luther-Universität Halle-Wittenberg, 06120 Halle, Germany

## Abstract

Our understanding of the properties of ferromagnetic materials, widely used in spintronic devices, is fundamentally based on their electronic band structure. However, even for the most simple elemental ferromagnets, electron correlations are prevalent, requiring descriptions of their electronic structure beyond the simple picture of independent quasi-particles. Here, we give evidence that in itinerant ferromagnets like cobalt these electron correlations are of nonlocal origin, manifested in a complex self-energy Σ_*σ*_(*E*,**k**) that disperses as function of spin *σ*, energy *E*, and momentum vector **k**. Together with one-step photoemission calculations, our experiments allow us to quantify the dispersive behaviour of the complex self-energy over the whole Brillouin zone. At the same time we observe regions of anomalously large “waterfall”-like band renormalization, previously only attributed to strong electron correlations in high-*T*_C_ superconductors, making itinerant ferromagnets a paradigmatic test case for the interplay between band structure, magnetism, and many-body correlations.

## Abstract (web summary)

The consequences of electron-electron interactions are difficult to calculate reliably but this is needed to understand important physical properties such as ferromagnetism. Tusche et al. show that interaction effects in cobalt are nonlocal, presenting a challenge to future theoretical approaches.

## Introduction

Today’s information technology vastly depends on ferromagnetic materials used not only for storage but also processing of information in prospective spintronic devices^1,2^. Designing new magnetic materials requires a thorough understanding of their electronic structure. Nevertheless, the common description of electrons in solids in terms of a band structure of independent quasi-particles is only of limited use, even for the most simple elemental ferromagnets. The reason is that the microscopic origin of ferromagnetism is based on the quantum mechanical exchange interaction between the electrons—a fundamental many-particle effect. Today, more refined models, accounting for the role of many-body interactions in solids, are just emerging.

A fundamental concept in solid state physics is the description of electrons in a crystal by their energy *E* and their momentum **k** in a band structure of independent quasi-particles. For many materials, in absence of strong correlations, the band features can be predicted successfully within the local density approximation (LDA) to density-functional theory (DFT)^3^. This is actually not a trivial finding, as it requires the interactions between electrons to be screened in a way that each electron propagates in an effective one-particle potential. The result is the well-known representation of electronic states in a diagram with infinitely sharp bands^4^. Many-body interactions which are only partially captured in this independent-particle picture lead to a renormalization of quasi-particle energies and to a finite lifetime. As a quasi-particle spends only a limited time in one state before being scattered, it follows from Heisenberg’s uncertainty principle that also its energy is not sharply defined. In a band structure representation this has a particular consequence: the bands become smeared out in energy and momentum, as lifetime is shortened. An experimental access to the spectral function, containing all information on quasi-particle renormalization and broadening, is provided by photoelectron spectroscopy. For prototypical systems, like the noble metal copper, remarkable agreement with calculated maps of the photocurrent was obtained^5^.

In ferromagnets, the same treatment results in a spin-split band structure with states of well-defined spin-up and spin-down character. This effective one-electron theory is often sufficient to describe the ground-state properties such as lattice constants or the shape of the Fermi surface of the elemental 3d ferromagnets^6^. In photoemission experiments, however, a clear separation of majority (↑) and minority (↓) spin bands is usually not observed. It was already recognized early that electron correlations, expressed by a complex self-energy Σ, are significant for all narrow-band 3d ferromagnets, even when the single-electron picture might lead to a coincidental agreement with photoemission experiments^7^. Since then, our basic understanding of strong correlations is based on the coulomb interaction between localized electron and hole states, and often can correctly describe many-body effects in core levels, but also the 6 eV satellite in valence band photoemission spectra from ferromagnetic Ni^8,9^.

While still being local, recent theoretical concepts like the dynamical mean-field theory (DMFT) also include dynamic correlations^10^. For ferromagnets, this leads to a pronounced spin-dependent broadening of the majority states below *E*_F_^11,12^. Nevertheless, spin-resolved photoemission experiments showed that such theories still fail for a quantitative description: they clearly underestimate the broadening as well as the energy renormalization in the valence band^13^. While the present theoretical treatment of electron correlations in the ferromagnet is not sufficient, it was assumed that a proper description might rely on additional nonlocal effects^13,14^. Their role in an itinerant ferromagnet, however, is still elusive.

Nonlocal correlations would be characterized by the interaction between many-particle wave functions, extending over several lattice constants instead of being bound to individual atomic sites. As such, the complex self-energy Σ, that quantifies the renormalization of the quasi-particle potential, needs to depend on the wave vector of the respective states. Up to now, Σ has only been treated as a function of spin *σ* and energy *E*^11,14^ but not of the wave vector **k**, which would be decisive for nonlocal effects^14,15^. This limitation is mainly imposed upon lack of comprehensive experimental data of the spin-dependent spectral function, in consequence of the notoriously low efficiency of spin-resolving experiments.

Here, we show that in itinerant ferromagnets like cobalt these electron correlations are of nonlocal origin, manifested in a complex self-energy Σ_*σ*_(*E*,**k**) that disperses as function of spin *σ*, energy *E*, and momentum vector **k**. This observation becomes possible by using momentum microscopy^16^ with imaging spin analysis^17^ to measure extensive maps of the spin-resolved spectral function. Together with one-step photoemission calculations, the combined approach allows us to quantify the dispersive behaviour of the self energy. At the same time we observe regions of anomalously large “waterfall”-like band renormalization, previously only attributed to strong electron correlations in high-T_*C*_ superconductors^18^, making itinerant ferromagnets a paradigmatic test case for the interplay between band structure, magnetism, and many-body correlations. As such this finding has consequences not only for magnetism but for all phenomena in solid state physics driven by electronic correlations.

## Results

### Spin-resolved electronic states of cobalt

**Fig. 1:**
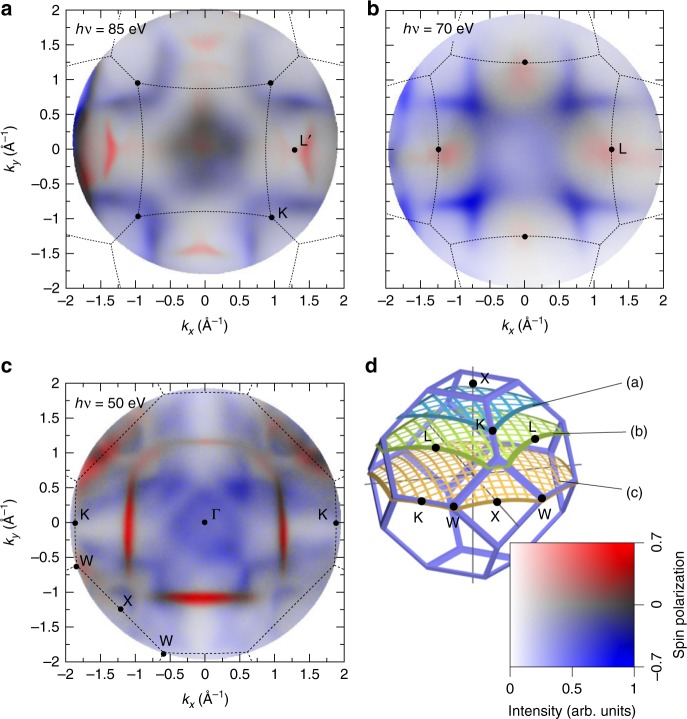
Measured spin-resolved Fermi surface of fcc cobalt. Spin-resolved photoemission intensities in selected sections through the three-dimensional Fermi surface of 18 ML Co/Cu(100) measured at photon energies of *hv* = 85 eV (**a**), *hv* = 70 eV (**b**), and *hv* = 50 eV (**c**). The boundary of the bulk fcc Brillouin zone is indicated in each section together with the high-symmetry points. Electronic features are repeated in neighbouring zones, as indicated for the points L′ and L that both belong to the “neck”-like connection of the majority FS at the (111) faces. **d** The fcc Brillouin zone together with the corresponding cuts sampled by **a**–**c**. According to the 2D colour code at the lower right, red and blue intensities correspond to majority and minority electronic states

Only recently, the long-standing problem of an efficient spin-resolved mapping of the spectral function has been solved by the advent of two-dimensional (2D) spin filters^17^, combined with momentum microscopy^16^ (see Methods). We carried out such measurements for thin films of the itinerant ferromagnet cobalt, at the Elettra synchrotron. Figure 1a–c shows the measured spin-resolved spectral function at the Fermi surface (FS) of the ferromagnet. The 2D momentum discs show tomographic sections through the face-centred-cubic (fcc) Brillouin zone (BZ) in reciprocal space (Fig. 1d), while the perpendicular momentum component (*k*_⊥_) is selected by the photon energy^19^. The spin-resolved momentum discs provide a comprehensive experimental access to the majority and minority spin contributions in the cobalt FS (see also Supplementary Fig. 1), both observed as sharp states.

In Fig. 1c, the photon energy (*hv* = 50 eV) was chosen such that we sample the centre (Γ point) of the BZ. At Γ, we find only minority spin states, and the majority FS sheet forms a sharp square-like contour. The weak majority intensity at the BZ boundary (W-X-W) originates from states 300 meV below *E*_F_. For further discussion of the quasi-particle self energy we will focus on this central section, which covers all possible radii, represented by the distance *k*_n_ between the Γ-point and the BZ boundary, within a single momentum disc. As we will see below, this is particularly useful as it leads to the largest variation of the self energy over the full 2D momentum disc.

**Fig. 2:**
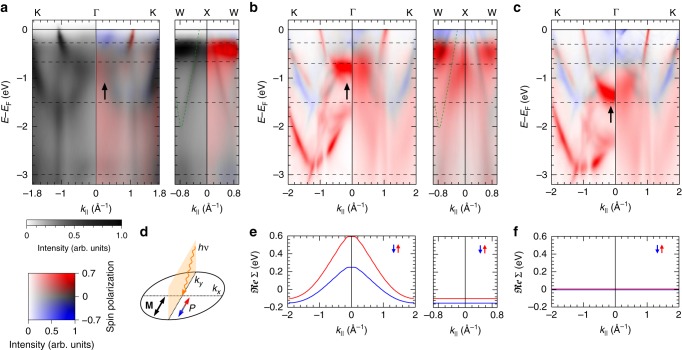
Spin-resolved band dispersion maps. **a** Dispersion of cobalt bands along the K-Γ-K and W-X-W directions as function of binding energy (*E*-*E*_F_), measured at *hv* = 50 eV. The left half in **a** shows the spin-averaging experiment on a grey intensity scale, and the right half the spin-resolved experiment in the same 2D colour code as in Fig. 1. Dashed horizontal lines indicate energies of 2D momentum maps in Fig. 4. **b** Calculated spin-resolved photoemission intensities corresponding to **a** using momentum-dependent corrections for the band renormalization $\frak{Re}$Σ_*σ*_(**k**) as in **e**, and constant (left half) or *E*-dependent broadening ℑ$\frak{m}$Σ_*σ*_(*E*) (right half). Dashed curves indicate faint band positions as guide to the eye. **c** Calculation using a constant $\frak{Re}$Σ for as in **f**. The bottom of the majority electron parabola (see solid arrows) lies too deep, compared with the experiment in **a** and calculation in **b**. The right half of panel **c** uses an energy depended ℑ$\frak{m}$Σ_*σ*_(*E*) following DMFT calculations from Ref. ^11^, which underestimates broadening close to *E*_F_. **d** Photoemission geometry used both, in experiment and theory: The photon beam is incident along the positive *k*_*y*_ direction with 25° towards the surface, magnetization **M** and polarization *P* are in-plane along *k*_*y*_

Figure 2a shows the energy dispersion of majority and minority spin features along the K-Γ-K and W-X-W directions. Unlike band diagrams often found in solid state physics text books, the majority band broadens already 300 meV below *E*_F_. The spin-resolved experiment (right half in Fig. 2a) reveals that the apparent background intensity is of majority spin character, and sets in together with the strong majority state broadening. In the W-X-W direction, this further coincides with a strong majority band feature, indicating that the presence of a high spectral density in the small (*E*,**k**) region gives rise to pronounced electron correlation^20^. By contrast, broadening of the minority spin bands is found to be considerably lower, and U-shaped features are observed down to *E*_F_–1.6 eV along Γ-K.

We observe additional non-dispersive vertical lines of intensity (see Fig. 2a) that extend along the energy axis. These “waterfall”-like intensities were previously observed in photoemission measurements of high-T_C_ superconductors^18^ and the effect was termed high-energy anomaly (HEA). The HEA was recently attributed to the presence of strong electron correlations connected with an anomalously large band renormalization^21,22^. It was shown that such waterfall intensities can also emerge as a result of photoemission matrix elements by the suppression of intensities along high-symmetry directions^23,24^. Here, we can rule out the latter explanation since the observed waterfalls are not in the vicinity of such suppressed intensities. Their observation in an itinerant ferromagnet provides now first evidence for the HEA being a general effect due to electron correlations. In this picture, waterfall intensities arise due to the short quasi-particle lifetimes in the ferromagnet. In particular, this results in imaginary valued wave vectors (see Methods) brought about by the complex self-energy potential^25^. As photoemission only probes the real wave vector component, $\frak{Re}$(**k**), vertical non-dispersive lines are observed in the experiment (see Supplementary Fig. 2).

### Complex self energy of the quasi-particle states

For a quantitative evaluation of electron correlations we performed photoemission calculations within the one-step-model (1SM: see Methods). In the 1SM, the complex self-energy Σ quantifies correlations in terms of a finite lifetime and energy renormalization relative to the LDA ground state. Photoemission intensities are calculated for the complete 2D momentum distributions, and therefore allow a direct comparison with measured momentum discs.

In weakly correlated electron systems, interactions are often well described within the GW approximation^3,20^. As it was shown for the non-magnetic metal copper, the variation of the self energy is sufficiently small such that wave functions of *d*- and *sp*-symmetry are well corrected by constant self energies $\frak{Re}$Σ_*d,sp*_, respectively^5^. This simplification can be understood as a very first step towards a more complete description of the electronic self-energy. Nevertheless, it still assumes that for each orbital wave function electron correlations in the metal are local, being connected to individual lattice sites, in contrast to the nonlocality of valence band wave functions themselves. This approach, however, represents too simple an approximation that does not lead to convincing results for materials in which the electron interaction is critical. Here, we give evidence that for the itinerant ferromagnet cobalt, the self energy needs to be considered as being nonlocal, such that Σ_*σ*_(*E*,**k**) shows a pronounced dispersion within the BZ.

In general, the self-energy Σ_*σ*_(*E*,**k**) is a complex number, written as the sum of its real- and imaginary-part, Σ_*σ*_(*E*,**k**)=$\frak{Re}$Σ_*σ*_(*E*,**k**)+i·ℑ$\frak{m}$Σ_*σ*_(*E*,**k**). Instead of considering the joint dependence on energy *E* and wave vector **k**, we assume for simplicity that $\frak{Re}$Σ_*σ*_(*E*,**k**) depends only on **k** and ℑ$\frak{m}$Σ_*σ*_(*E*,**k**) depends only on *E*, leading to Σ_*σ*_(*E*,**k**)=$\frak{Re}$Σ_*σ*_(**k**)+i·ℑ$\frak{m}$Σ_*σ*_(*E*). The imaginary part leads to damping of the wave functions, and is therefore associated with a short lifetime of the quasi-particle state. When we use a small constant imaginary self energy (ℑ$\frak{m}$Σ = 0.05 eV) all states appear as relatively sharp bands in the calculation, as shown in the left half of Fig. 2b, c. Especially an appearance of the U-shaped majority states below *E*_F_-2 eV follows the classical text book picture of exchange splitting, but these states are completely smeared out in the experiment.

**Fig. 3:**
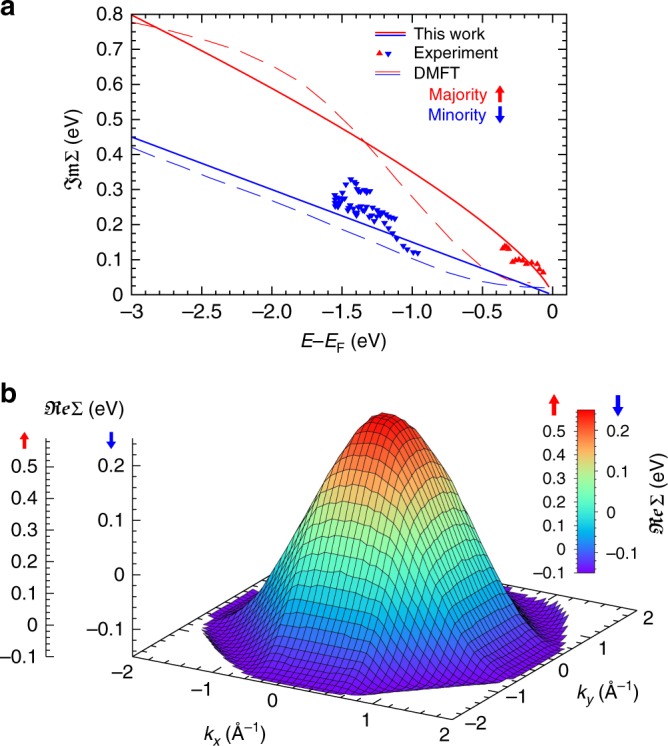
Complex self-energy in dependence of energy and momentum in the BZ. **a** Imaginary part of the spin-resolved self energy as a function of the binding energy. Symbols show data obtained from the line widths of majority and minority bands from our experiment. Dashed lines show the lifetime broadening from previous work using DMFT^11^, and solid lines show the functional dependence that leads to the best agreement of the experiments with our photoemission calculations. **b** Variation of the quasi-particle renormalization $\frak{Re}$Σ_*σ*_(**k**) in the (*k*_*x*_*,k*_*y*_) plane cutting through the BZ centre. Renormalization of minority (↓) and majority (↑) states depends only on the distance |**k**| from the Γ point to the BZ border, where $\frak{Re}$Σ_*σ*_(**k**) becomes constant and periodic boundary conditions are fulfilled

In a first step, we therefore describe the strong broadening of majority spin states that is observed in Fig. 2a to set in at about 300 meV below *E*_F_ by a spin- and energy-dependent imaginary part of the self-energy ℑ$\frak{m}$Σ = ℑ$\frak{m}$Σ_*σ*_(*E*). The corresponding energy dependence of ℑ$\frak{m}$Σ that is used in our calculations is shown in Fig. 3a for both spins.

The functional dependence for majority and minority spin electrons was chosen such that best agreement of the calculated photoemission intensities is obtained with the measured spin-resolved spectral function as shown in Fig. 2a, as well as in the full 2D momentum discs at several binding energies. This ℑ$\frak{m}$Σ_*σ*_ follows qualitatively previous DMFT findings^11^, which however underestimated the broadening, particularly close to *E*_F_ (see Fig. 3a). The noticeably increased broadening found directly below *E*_F_ is further confirmed by line widths (symbols) extracted from individual spectra (see Supplementary Fig. 3).

### Dispersion of the self energy in the full BZ

The *E*-dependent ℑ$\frak{m}$Σ alone is not sufficient to fully describe the experimental momentum discs and spectral function. This becomes most obvious by looking at the position of the bottom of the parabolic majority band at Γ that lies at too large binding energy in Fig. 2c. We note that also including the energy-dependent broadening according to ref.^11^ is not sufficient to shift majority intensity to the onset of the majority band bottom indicated by the dashed line at *E*_F_ –0.7 eV.

In order to include the **k** dependency of the renormalization, we describe $\frak{Re}$Σ = $\frak{Re}$Σ_*σ*_(**k**) as a smooth function that only depends on the distance from the BZ centre, as displayed in Fig. 3b (see Methods). The measured spin-resolved momentum discs are particularly sensitive to the energy position of majority and minority spin states. A direct comparison with the calculated ones therefore allows to test the renormalization all over the entire BZ. The effect of the **k**-dependent renormalization is shown in Fig. 2b, where the states close to Γ are corrected upwards, while the energy agreement for states at the BZ border is preserved. The corresponding mass renormalization m*, expressed by the flattening of the bands, is an important signature of electron correlations^26^. In particular, spectral features along the W-X-W direction are perfectly reproduced, such as the majority intensities at *E*_F_-300 meV and the faint minority states dispersing down to *E*_F_-2 eV (compare dashed curves in Fig. 2a, b).

We also tested the effect of a purely local treatment of correlations which would lead to a description of the self energy only dependent on energy, but not on wave vector. While such a self energy of the form Σ_*σ*_(*E*) = $\frak{Re}$Σ_*σ*_(*E*) + i·ℑ$\frak{m}$Σ_*σ*_(*E*), as used in previous work, can indeed lead to similar photoemission spectra at a single **k** point, i.e., the Γ point, the renormalization of states at large **k**, i.e., at the W-X-W line, is not described well. In a constant energy momentum disc, states likewise would intersect at different *k*_||_, leading to qualitatively different photoemission patterns than observed in the experiment. Therefore, a model of only local correlations can not describe the self energy, and thus the renormalization of states equally well as found for the wave-vector-dependent self energy, reported here (see Supplementary Fig. 5 for a comparison). This is consistent with findings from refs.^13,14^, where the DMFT approach—as a local theory—was not able to reproduce the correct band energies at all wave vectors.

**Fig. 4:**
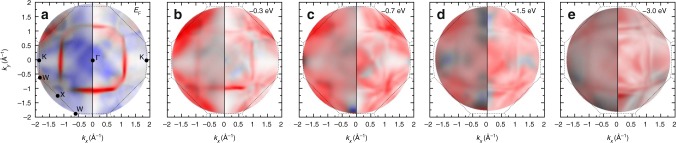
Spin-resolved photoemission momentum maps. **a**–**e** Selected constant energy momentum discs from the spin-resolved experiment (left half of each panel) for *hv* = 50 eV at *E*_F_ (**a**) to *E*_F_-3.0 eV (**e**). These sections are also marked in Fig. 2. Calculated photoemission maps at the same energies are displayed on the right half of each image. The calculations take into account the energy and momentum-dependent corrections for ℑ$\frak{m}$Σ_*σ*_(*E*) and $\frak{Re}$Σ_*σ*_(**k**). Red and blue colours correspond to the intensities of majority and minority electronic states, respectively

The complex self energy as described above is a function of *E*, **k**, and the electron spin. This Σ_*σ*_(*E*,**k**), needs to simultaneously describe the effect of many-body induced lifetime broadening and renormalization over the entire BZ and for all energies *E*-*E*_F_. A sensitive test if this description is successful is the comparison of measured spin-resolved 2D momentum discs with the corresponding theoretical ones^5^. Experimental momentum discs are shown in Fig. 4a–e side-by-side with the result from the 1SM calculations in a wide energy range. We find a remarkably good agreement of all prominent features in the complete energy and momentum range, confirming the complete self-energy dispersion Σ_*σ*_(*E*,**k**) including the energy-dependent broadening ℑ$\frak{m}$Σ_*σ*_(*E*) and the wave-vector-dependent renormalization $\frak{Re}$Σ_*σ*_(**k**).

The spin-dependent broadening, being much larger for majority electrons, leads to quantitative agreement of the theoretical spectrum with the experiment, reproducing sharp bands and the FS-contour at *E*_F_ and the onset of a featureless majority spin background. In the FS-contour, even tiny features, like the rings of minority states around the X point and the pattern around the Γ-point are quantitatively reproduced, compared with the experiment in Fig. 1c. As for the energy broadening, renormalization is found to be much stronger for majority spin states than for the minority ones.

**Fig. 5:**
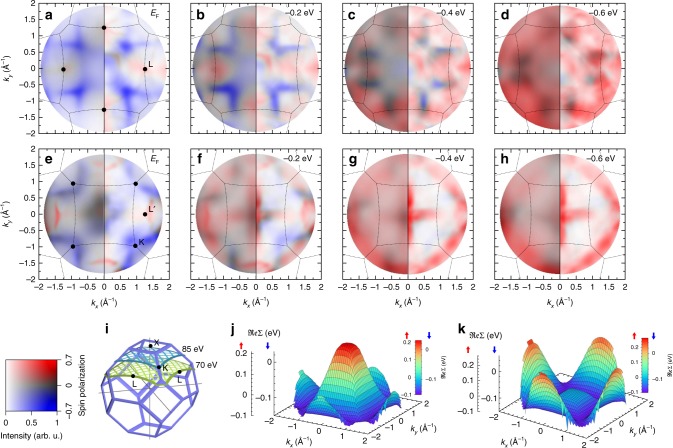
Spin-resolved photoemission momentum maps at *hv* = 70 eV and 85 eV. Selected constant energy images from the spin-resolved experiment (left half) for *hv* = 70 eV (**a**–**d**), and for *hv* = 85 eV (**e**–**h**). While momentum discs at these *hv*s (**i**) do not intersect with the high majority spectral density along the W-X-W directions, also here a pronounced increase of majority background intensity is observed between −0.2 eV and −0.4 eV, indicating the particularly short majority state lifetime few 100 meV below *E*_F_. Position and broadening of majority and minority features are well reproduced in the calculated photoemission maps (right half). Calculations take into account the energy and momentum-dependent corrections for ℑ$\frak{m}$Σ_*σ*_(*E*) and $\frak{Re}$Σ_*σ*_(**k**). **j** Variation of $\frak{Re}$Σ_*σ*_(**k**) as function of *k*_||_ at *hv* = 70 eV. **k** The same for *hv* = 85 eV

The same model also applies for wave vectors with *k*_⊥_ ≠ 0. To verify this behaviour, we also compare the results from the 1SM with spin resolved momentum discs measured at photon energies of 70 eV and 85 eV. As outlined in Fig. 1, those sections do not run through the Γ-point. The results are shown in Fig. 5a–d, e–h, respectively. The 1SM calculations displayed in the right half of each panel uses the same functional dependence for Σ_*σ*_(*E*,**k**), as above. However, as the corresponding wave vectors are offset along the *k*_⊥_ axis from the Γ-point, there is a minimum distance |**k**| from Γ, even in the centre of the momentum disc. As Σ_*d*_(**k**) is evaluated in the three-dimensional (3D) BZ, the amplitude is therefore reduced compared to Fig. 3b at 50 eV. Likewise, moving to the adjacent BZ (compare dotted lines in Fig. 5a–h) results in an increased value of Σ, since those wave vectors have a *k*_⊥_ in their respective BZs.

Also for the momentum discs intersecting the BZ above the Γ-point an excellent quantitative agreement of the photoemission patterns between experiment and the 1SM is found: in particular, calculated photoemission patterns at the same time show agreement with the experiment both, in the centre (*k*_||_ ≈ 0) and at the outer regions of the momentum discs, e.g., at the L or L’ points in Fig. 5, confirming the variation of $\frak{Re}$Σ over the full 3D BZ.

## Discussion

To this end, we separated the description of the complex self energy to the imaginary part (describing the broadening) ℑ$\frak{m}$Σ_*σ*_(*E*) which depends on the binding energy, and to the real part (describing the renormalization) $\frak{Re}$Σ_*σ*_(**k**), which depends on the wave vector. This description represents the most basic model that is able to reproduce measured photoemission intensities.

A momentum disc always shows a section through the reciprocal space at constant energy, and therefore allows a direct access to the **k**-dependent self energy. When correlations are treated locally, without **k**-dependence, it was found that an energy dependent $\frak{Re}$Σ_*σ*_(*E*) would lead to an improved general agreement with experiments^11,27^. However, this is not sufficient to explain quantitatively a different renormalization as function of the wave vector, but at constant *E*, as is observed here. As the same $\frak{Re}$Σ_*σ*_(**k**) describes the renormalization at energies between *E*_F_ and *E*_F_-3.0 eV, we conclude that an explicit energy dependence plays only a minor role, and the predominant correlations in cobalt are nonlocal. An additional energy dependence expressed in a joint $\frak{Re}$Σ_*σ*_(*E*,**k**), however, becomes important for states far below *E*_F_^3,5,20^.

Likewise, the nonlocal origin of correlations is also expected to lead to a momentum-dependent correction of the lifetime broadening. This momentum dependence in the form ℑ$\frak{m}$Σ_*σ*_(*E*,**k**) is shown in Supplementary Fig. 4 for experimental line widths obtained from the photoemission measurement at different points in the BZ. The figure reveals that indeed states at the same binding energy *E*, but at a different distance from the BZ centre have a different magnitude of ℑ$\frak{m}$Σ_*σ*_. Form Supplementary Fig. 4, this variation over the BZ zone is in the order of 20–30 %, leaving the energy dependence the dominating contribution. Nevertheless, the nonlocal correlations lead to a discernible wave vector dependence of also the imaginary prat of the self-energy in the form ℑ$\frak{m}$Σ=ℑ$\frak{m}$Σ_*σ*_(*E*,**k**).

Commonly, correlation is understood as a localization of charges to lattice sites in real space. Consequently, such localization leads to electronic states that do not disperse in energy over the reciprocal space. Examples are found in compounds of the heavy rare earth elements where strongly localized f-electron states are responsible for the weakly dispersing heavy fermion bands but also for the core levels of the elemental ferromagnets, for which spin-dependent renormalization was firstly observed^28,29^. By contrast, the 3d-electron states responsible for band ferromagnetism are only weakly localized, and characterized by a sizeable energy dispersion over the BZ. Nevertheless, these electronic states are dominated by spin-dependent many-body effects, putting ferromagnets like cobalt at the border to the large class of strongly correlated materials. Advanced theories that treat nonlocal correlations in transition metals are just emerging, and need substantial quantitative input from detailed experiments^26^. The finding that the complex self-energy Σ_*σ*_(*E*,**k**) likewise exhibits a dispersion as function of the wave-vector **k** underlines the nonlocal character of the electron correlations, and substantially affects our understanding of many-body electron interactions in solids, in general.

## Methods

### Spin-resolved momentum microscopy

Spin- and momentum-resolved photoelectron spectroscopy experiments were carried out at the NanoESCA beamline^30^ of the Elettra synchrotron in Trieste (Italy), using p-polarized photons in the energy range between 50 eV and 85 eV. All measurements were performed while keeping the sample at room temperature. Photoelectrons emitted into the complete solid angle above the sample surface were collected using a momentum microscope^31^. The momentum microscope directly forms an image of the distribution of photoelectrons as function of the lateral crystal momentum (*k*_*x*_,*k*_*y*_) that is recorded on an imaging detector. Here, we refer to these 2D intensity maps as “momentum discs”, representing constant energy cuts through the valence band spectral function I(*k*_*x*_,*k*_*y*_,*k*_⊥_,*E*), whereas the perpendicular momentum component is fixed by photoemission selection rules (see below).

An imaging spin filter based on the spin-dependent reflection of low-energy electrons at a W(100) single crystal^17^ allows the simultaneous measurement of the spin polarization of photoelectrons in the whole surface BZ. Images were recorded after reflection at a scattering energy alternating between 26.5 eV and 30.5 eV, switching the spin sensitivity *S* of the detector between 42% and 5%, respectively. From these images, the spin polarization at every (*k*_*x*_,*k*_*y*_) point in a momentum disc (e.g., see Fig. 1a–c) is derived as $P\left( {k_x,k_y} \right) = \left[ {{\cal I}_{{\mathrm{26}}{\mathrm{.5eV}}}\left( {k_x,k_y} \right) - {\cal I}_{{\mathrm{30}}{\mathrm{.5eV}}}\left( {k_x,k_y} \right)} \right]/\left[ {S_{{\mathrm{26}}{\mathrm{.5eV}}} \cdot {\cal I}_{{\mathrm{30}}{\mathrm{.5eV}}}(k_x,k_y) - S_{{\mathrm{30}}{\mathrm{.5eV}}} \cdot {\cal I}_{{\mathrm{26}}{\mathrm{.5eV}}}\left( {k_x,k_y} \right)} \right]$, where ℐ_ES_ denotes the measured intensity image at scattering energy ES normalized by the respective reflectivity as measured from the clean Cu(100) surface^32^. A series of measurements results in a spin-resolved 3D data set *I*_*σ*_(*k*_*x*_,*k*_*y*,_*E*), from which the spectral function along arbitrary sections E(*k*_||_) can be extracted. Figure 2a shows such sections along different directions in the BZ.

The W(100) crystal of the spin filter was prepared by several flashing cycles in oxygen atmosphere, followed by a high temperature (2400 K) flash. This standard procedure is known to lead to clean, carbon free tungsten surfaces^32^. At a pressure of 1·10^−10^ mbar inside the spin-filter chamber during the measurements, the analyser-crystal could be used for 2 hours, before a degradation of the image was observed. This time frame allowed for collecting spin-resolved momentum patterns at several energies.

### Spin-resolved FS tomography

Images recorded by the momentum microscope represent 2D sections through the electronic structure of the sample. When the initial state is tuned to *E*_F_, i.e., selecting the electron kinetic energy *E*_kin_ = *hv*−*Φ*_A_, *Φ*_A_ being the work function of the energy analyser, this section runs through the FS of the sample. While the crystal momentum (*k*_*x*_,*k*_*y*_) in the surface plane is in general conserved in photoemission as result of the in-plane translation symmetry—and directly displayed in the momentum disc—this does not strictly apply to the component perpendicular to the surface. However, *k*_⊥_ can be selected by the photon energy, such that initial states in the BZ lie on a sphere of constant momentum^33^, representing a tomographic section through the BZ^19^. The radius is given by $\left| {\mathbf{k}} \right| = \frac{{\sqrt {2m} }}{h}\sqrt {E_{{\mathrm{kin}}} + V_{\mathrm{0}}}$. The value of the inner potential, *V*_0_, is in general a material-dependent quantity. Here, we determined *V*_0_ experimentally from a series of constant initial state (CIS) momentum maps. From the CIS scan, we determined a value of *V*_0_ = 17 eV, and *hv* = 50 eV for the section through the centre of the BZ.

For a qualitative understanding of the general appearance and shape of different bands in the cobalt FS, we refer to the 3D band calculation in Supplementary Fig. 1. Close to the upper X point (centre of Fig. 1a), only the area inside the central square lies within the first BZ in lateral direction. There, overlapping majority and minority states lead to a very-low total spin polarization of photoelectrons (grey colours correspond to zero polarization). We note that this area of the BZ is typically probed at low photon energies close to the emission threshold^34^, where only low spin polarization at *E*_F_ is generally observed in direct photoemission^35^.

At the L points, centred at the hexagonal (111) faces of the BZ (see Fig. 1b), we find majority states connecting the neighbouring BZs, similar to the “necks” of the FS in noble metals like copper, while the minority FS sheet has most intensity in the corners. This connection at the L point is also observed in Fig. 1a, where the section runs slightly above the L point (labelled L’), and the intensity is shifted towards the strong triangular feature that belongs to the majority FS sheet of the next BZ. This demonstrates that the sections (a)-(c) cover a *k*_⊥_ slice of finite thickness, due to the finite probing depth at these photon energies.

### Growth of ultra-thin Co films

Face-centred-tetragonal (fct) cobalt films with a thickness of 18 monolayers (ML) were deposited by electron beam evaporation onto a clean Cu(001) single crystal at a substrate temperature of 400 K. Before deposition, the Cu(001) single crystal was cleaned in-situ by cycles of Ar-ion sputtering and annealing. The epitaxial growth on the Cu(001) substrate stabilizes the cobalt film in the cubic phase, while the out-of-plane lattice direction experiences a small tetragonal distortion. For all theoretical calculations, the interlayer distances were chosen as *d*_12_ = 1.72 Å for the first, *d*_23_ = 1.74 Å for the second, and *d*_34_ = 1.73 Å for the third and all deeper Co layers, according to experimental results^36^. Before carrying out spin resolved momentum microscopy measurements, the samples were uniformly magnetized along the quantization axis of the spin filter (see Fig. 2d).

### Photoemission theory

The calculations of photoemission intensities rely on the local spin-density approximation (LSDA) to DFT, using a relativistic multiple-scattering approach (layer-Korringa-Kohn-Rostoker (LKKR)) as implemented in the OMNI code (see Code Availability). The calculations were performed for semi-infinite systems which consist of an fcc Cu substrate and 18 layers of Co. The photoemission spectra were computed within the one-step model (1SM). Lifetime effects were included by an imaginary part, ℑ$\frak{m}$Σ, of the energy, where the dependence on *E* from Fig. 3a was included for majority and minority states, respectively. Its dependence has been chosen to achieve the best possible agreement between the energies of quasi-particle bands within the experimental spectra and the calculated photoemission results. For the unoccupied states, we took ℑ$\frak{m}$Σ = 1.0 eV for majority and 3.0 eV for minority states.

Following previous results for the Cu substrate^5^, we include orbital-dependent corrections to the self energy by shifting the LSDA potentials of Cu by $\Sigma _d^{{\mathrm{Cu}}}$ = −0.8 eV for *d*-states and $\Sigma _{sp}^{{\mathrm{Cu}}}$ = + 0.3 eV for *sp*-states. For the cobalt states, this approach is not sufficient due to considerably stronger electron correlation. Being a nonlocal operator, Σ depends on energy, wave-vector **k**, atomic layer and angular momentum. Here, we include the additional functional dependence of $\frak{Re}$Σ(|**k**|) on the distance |**k**| from the Γ point, as displayed in Fig. 3b.

### Wave-vector-dependent self energy

The description of the functional-dependence of $$\frak{Re}$$Σ(**k**) was chosen to depend on as few parameters as possible. For a simultaneous description within the 3D reciprocal space by the same model, the momentum dependence has to fulfil several requirements. These are the invariance with respect to the symmetry operations of the bulk BZ, the continuity of the self energy, and a vanishing first derivative at mirror planes of the reciprocal lattice which are present on every face of the bulk BZ. To this end, we introduced a normalized coordinate $$k_{\mathrm{n}}\left( {\mathbf{k}} \right) = \left| {\mathbf{k}} \right|/\left| {{\mathbf{k}}_{{\mathrm{BZ}}}\left( {\mathbf{k}} \right)} \right|$$, where **k**_BZ_(**k**) is a vector to a point on the BZ border and is collinear to **k**. By this construction we get *k*_n_ = 0 at Γ and *k*_n_ = 1 everywhere on the border of the BZ. Then, $$f\left( {k_{\mathrm{n}}} \right) = \Sigma _\Gamma + \left( {\Sigma _{{\mathrm{BZ}}} - \Sigma _\Gamma } \right)\left[ {\left( {1 - {\mathrm{cos}}\left( {k_{\mathrm{n}}} \right)} \right)/2} \right]$$ evaluates to Σ_Γ_ at Γ and to Σ_BZ_ everywhere on the BZ border. In the vicinity of the Γ point, we use a more isotropic behaviour by $$k_{{\mathrm{iso}}}\left( {\mathbf{k}} \right) = \left| {\mathbf{k}} \right|/k_{{\mathrm{BZ,avg}}}$$, where *k*_BZ,avg_ is an average value of the distance Γ to the BZ border. The self-energy function then interpolates between the isotropic behaviour at Γ and the BZ adapted dependence near the BZ border: $$\frak{Re}$$$$\Sigma \left( {\mathbf{k}} \right) = f\left( {k_{\mathrm{n}}} \right)\left[ {1 - k_{\mathrm{n}}} \right] + f\left( {k_{{\mathrm{iso}}}} \right)k_{\mathrm{n}}$$. In this model, two free parameters, Σ_Γ_ and Σ_BZ_, were chosen separately for each spin: Σ_Γ,↑_ = 0.7 eV, Σ_BZ,↑_ = −0.1 eV, Σ_Γ,↓_ = 0.31 eV, Σ_BZ,↓_ = −0.15 eV. The self-energy function is defined in three-dimensional reciprocal space. To evaluate the self energy for the different sections probed by photoemission we determined the *k*_⊥_ coordinate from the free-electron model, as outlined above.

### High-energy anomalies and complex band structure

Here, we present an interpretation of the “waterfall” features, observed in our experiments (Fig. 2a), in terms of evanescent states that acquire their characteristic appearance in a complex-valued potential with a non-zero imaginary component.

To demonstrate the effect of complex-valued potentials on electronic dispersions, we start from the nearly-free electron model^37^ which generates a two-band dispersion with a band gap at the border of the BZ by using a single-frequency, cosine-type potential of the form *V*(*r*) = *V*_0_+*V*_g_cos(*gr*) for the ionic potential (*g* being the distance between two neighbouring points in the reciprocal lattice). The resulting band dispersion for the case ℑ$\frak{m}$*V*(*r*) = 0 is shown in Supplementary Fig. 2a, plotted as a function of complex-valued wave vectors. Electronic states with a non-zero imaginary part of the wave vector, known as evanescent states with finite spatial extension, are present in the band gap.

In Supplementary Fig. 2b, *V*_0_ has a negative imaginary part. By comparison to the Schroedinger equation for a quasi-particle^38^, ${\cal H}\psi + {\int} {\mathrm{d}}{\mathbf{r}}\prime \Sigma \left( {r,{\mathbf{r}}\prime ,E} \right)\psi \left( {{\mathbf{r}}\prime } \right) = E\psi$, this *V*_0_ can be reinterpreted as a self-energy of the form $\Sigma \left( {{\mathbf{r}},{\mathbf{r}}\prime ,E} \right) = \Sigma _0\delta \left( {{\mathbf{r}} - {\mathbf{r}}\prime } \right)$, where Σ_0_ has a negative imaginary part. Supplementary Fig. 2b thus represents a band dispersion under the influence of a limited lifetime of a quasi-particle state, such as scattering by other electrons.

A photoemission experiment probes the projection *E*($\frak{Re}$(***k***_||_)) onto the real coordinate axis. In this projection, dispersive bands in the complex space can appear as steep dispersive branches, similar to the vertical waterfall features observed in the experiment. Furthermore, all electronic states acquire an imaginary component in the wave vector, meaning their spatial extension is limited. Thereby, the spatial damping of states in the band gap is reduced relative to those in the allowed energy region. This may facilitate the observation of such in-gap electronic states in surface sensitive photoemission experiments.

The interpretation presented here requires no specific mechanism to the origin of waterfall features. They can therefore be considered a general phenomenon that might be observed in any material where the quasi-particle states are subject to short lifetimes. For instance, waterfall-like effects due to the complex band-structure were previously observed in theoretical studies of low-energy electron diffraction at a W(110) surface in ref. ^25^.

### Code availability

The computer code OMNI - Fully relativistic electron spectroscopy calculations by J. Henk et al. is available from the authors.

## Electronic supplementary material

Supplementary Information

## Data Availability

The data that support the findings of this study are available from the corresponding author upon reasonable request.
