# Supplementary material for: Nonlocal electron correlations in an itinerant ferromagnet
Source: Nat Commun. 2018 Sep 13;9:3727. doi: 10.1038/s41467-018-05960-5 (PMC6137183; doi:10.1038/s41467-018-05960-5)
Supplement: Supplementary file 1 — Supplementary Information [file 41467_2018_5960_MOESM1_ESM.pdf]

Supplementary Information:
Nonlocal electron correlations in an itinerant ferromagnet

Tusche *et al.*

SUPPLEMENTARY FIGURES

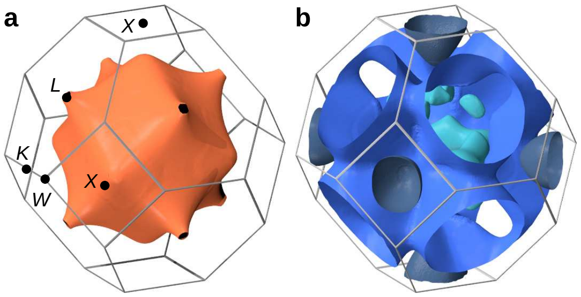

Supplementary Figure 1. **Calculation 3D Fermi surface of cobalt.** Spin-resolved FS of *fcc* cobalt, showing majority electron states (**a**), and minority electrons states (**b**). Labels in **a** indicate high symmetry points in the BZ (compare Fig. 1d). The majority FS sheet is formed by one band, characterized by a small 'neck' connecting adjacent BZs at the L points, as observed experimentally in Figs. 1a and 1b. The section though the BZ centre (Γ point) results in the square shaped feature observed experimentally at $h\nu$=50 eV (see Fig. 1c). The minority FS sheet contains three orbital contributions (indicated by light, solid, and dark blue colours), giving rise to various minoriy photoemission features in Figs. 1a-c.

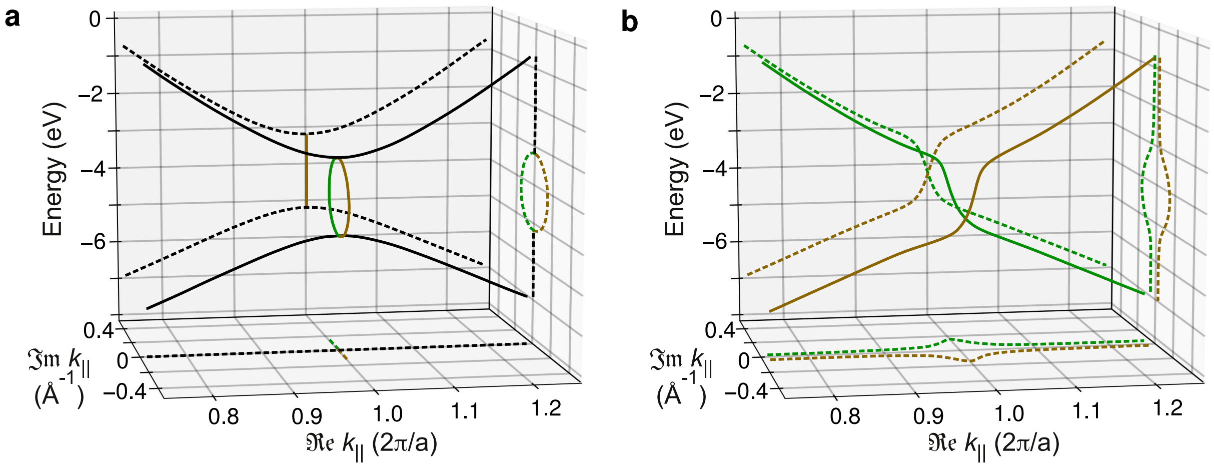

Supplementary Figure 2. **Nearly-free electron model with complex band structure.** **a**, Real-valued potential. **b**, Complex potential with a small imaginary part. Electronic states with real-valued wave vectors are plotted as black lines. Solid lines are curves within the 3D coordinate system. Dotted lines are two-dimensional projections. The $E(\mathfrak{Re}\, k_{||})$ projection is closest to the appearance in photoemission, which resolves the real-valued wave vector components.

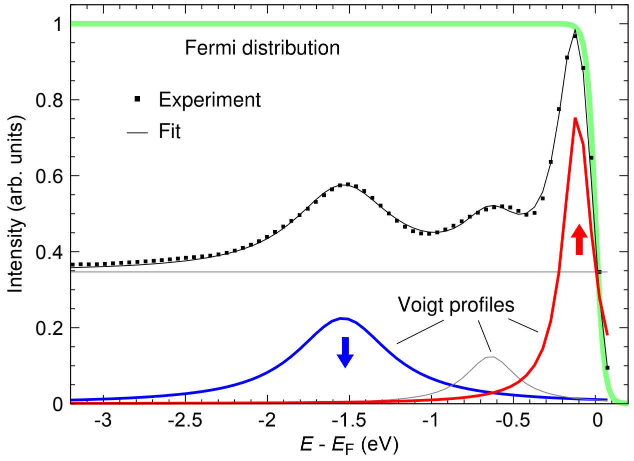

Supplementary Figure 3. **Majority and minority state linewidth.** Experimental intensity profile for the spin integrated experiment (solid points) at $k_z \approx 0$ ($h\nu$=50eV), k_y=0, and k_x=-1.0 Å^{-1} (e.g., see Fig. 2a). Majority (↑) and minority (↓) states were identified from the spin-resolved measurements and described by Voigt profiles, whereas the lifetime broadening and the instrumental resolution corresponds to the Gaussian and Lorentzian width parameters, respectively. Lifetime broadening for states at different energies (i.e., different k_x) are shown as solid points in Fig. 3a, and used as starting parameters for the spin-dependent $\mathfrak{Im}\Sigma_\sigma(E)$ used in the 1SM calculations.

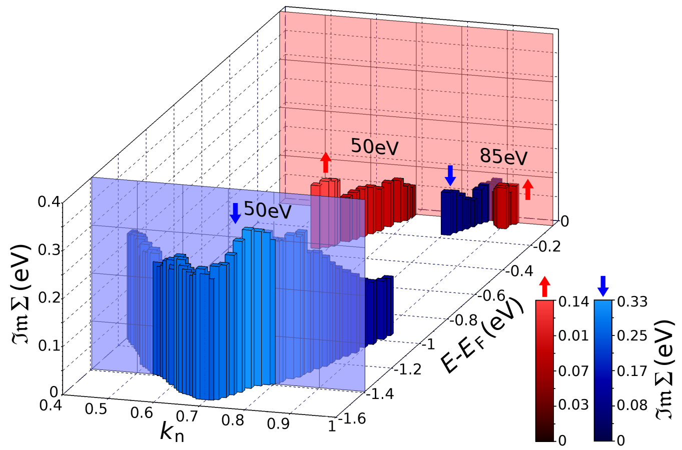

Supplementary Figure 4. **Dispersion of $\mathfrak{Im}\Sigma_\sigma$ over the BZ.** Experimentally obtained imaginary part of the self-energy $\mathfrak{Im}\Sigma_\sigma$ displayed as a function of binding energy, E, and the momentum coordinate k_n in the bulk BZ. Values of $\mathfrak{Im}\Sigma_\sigma(E, k_n)$ were obtained from the respective line width analysis as shown in Supplementary Fig. 3 for majority (↑) and minority (↓) states. As indicated in the Figure, data points cover the centre of the bulk BZ ($k_z = 0$) measured at $h\nu$=50eV, and distinct majority and minority bands close to the L-point measured at $h\nu$=85eV. The latter points are located close to the BZ boundary, corresponding to $k_n \approx 1$. Constant energy sections through the minority bands (blue plane) and majority bands (red plane) indicate that states with the same energy E, but different k_n have a different lifetime broadening $\mathfrak{Im}\Sigma_\sigma$ (0.24 eV vs. 0.32 eV for minority states at $E = -1.4$ eV, and 0.06 eV vs. 0.08 eV for majority states at $E = -0.07$ eV).

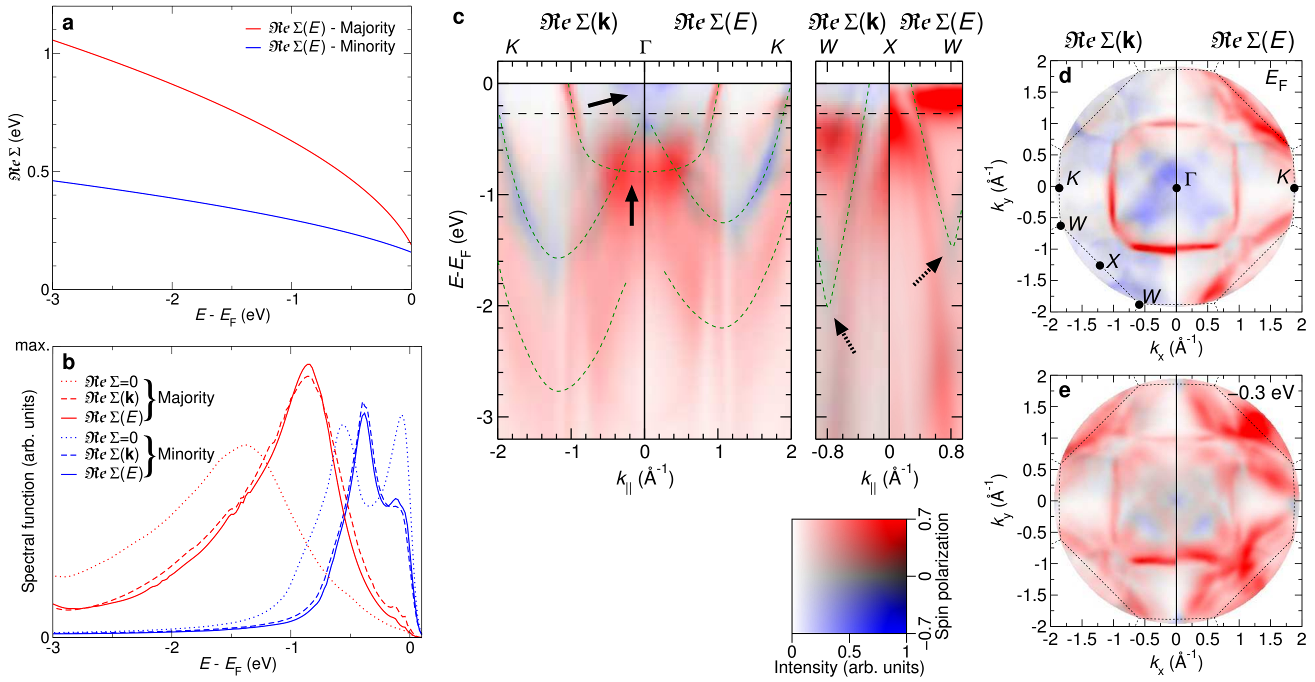

Supplementary Figure 5. **Effect of wave-vector vs. energy dependence of $\mathfrak{Re}\Sigma_\sigma$.** **a**, Model of an energy dependent $\mathfrak{Re}\Sigma_\sigma = \mathfrak{Re}\Sigma_\sigma(E) = a\sqrt{E-b}$ fitted such that renormalization of states at the Γ point (**b**) agrees with $\mathfrak{Re}\Sigma_\sigma = \mathfrak{Re}\Sigma_\sigma(\mathbf{k})$. **c**, Calculated photoemission intensities using $\mathfrak{Re}\Sigma_\sigma = \mathfrak{Re}\Sigma_\sigma(\mathbf{k})$ (left half) and $\mathfrak{Re}\Sigma_\sigma = \mathfrak{Re}\Sigma_\sigma(E)$ (right half). Dashed curves indicate band positions as a guide to the eye. Using the energy dependent self-energy form **a**, majority and minority state intensities close to the Γ point and the parabolic majority band close to E_{F} agree between both models (see solid arrows). Differences become pronounced with increasing $k_{||}$ for the U-shaped minority and majority bands. Along the W-X-W direction, at the BZ border, $\mathfrak{Re}\Sigma_\sigma(E)$ leads to strong majority intensity appearing already at E_{F} compared to E=-0.3 eV. In addition, the weak minority band (see dashed arrows), are shifted upwards by about 0.3 eV, in disagreement with the experiment (compare Fig. 2a). **d-e**, Constant enregy momentum discs at E_{F} and E_{F}-0.3 eV show the same photoemission patterns around the centre, but qualitative differences at the BZ border.
